# Interferon-γ +874T/A polymorphism, *Toxoplasma gondii* serostatus, and risk of paranoid schizophrenia in males: a case–control study

**DOI:** 10.3389/fpsyt.2026.1771815

**Published:** 2026-03-26

**Authors:** Dotan Braun, Ahikam Olmer

**Affiliations:** The Jerusalem Mental Health Center, Jerusalem, Israel

**Keywords:** +874T/A, gene–environment interaction, interferon-gamma, males, schizophrenia, toxoplasma gondii

## Abstract

**Background and Hypothesis:**

*Toxoplasma gondii* (TG) exposure and host immunogenetic variation have been implicated in psychosis. Interferon-gamma (IFN-γ) is central to TG control; the IFN-γ +874T/A polymorphism has been linked to variability in IFN-γ production. We hypothesized that TG seropositivity and IFN-γ +874T/A jointly increase risk for paranoid schizophrenia in males.

**Study design:**

We conducted a case–control study of adult males (103 cases meeting DSM-IV criteria for paranoid schizophrenia; 102 healthy controls) from two mental health centers in Israel. TG status was assessed serologically using automated ELFA/VIDAS assays. IFN-γ +874T/A was genotyped with Applied Biosystems TaqMan assays. Logistic regression modeled case status as a function of TG serostatus, genotype (AA/TA/TT), their interaction, and age. Hardy–Weinberg equilibrium (HWE) was tested among controls. Sensitivity analyses explored dominant, recessive, and additive genetic encodings.

**Study results:**

TG seropositivity rates were nearly identical in cases and controls. Genotype distributions among controls conformed to HWE. In the primary model, TG serostatus was not associated with case status, and no TG×genotype interaction was detected. Findings were consistent across sensitivity analyses.

**Conclusions:**

In this male paranoid-schizophrenia cohort, we found no evidence that TG serostatus, IFN-γ +874T/A, or their interaction contribute to disease risk. The near-identical TG+ prevalence across groups argues against large interaction effects under the exposure and measurement definitions used here. Transparent null results refine plausible effect sizes and caution against overinterpreting adult TG seropositivity as a biomarker of psychosis risk. Future studies should target developmental timing and immune-functional markers before infection-related screening or prevention can be justified.

## Introduction

Schizophrenia is a chronic, severe psychiatric disorder affecting ~1% of the global population and ranking among the leading causes of years lived with disability (1). It is characterized by delusions, hallucinations, disorganized thought and behavior, and marked impairment in social and occupational functioning. Despite decades of research, etiological understanding remains incomplete. Contemporary models emphasize a multifactorial origin involving genetic liability and environmental exposures across development. Twin and adoption studies estimate heritability at ~70–80%, yet monozygotic concordance well below 100% underscores non-genetic contributors. Genome-wide association studies implicate hundreds of common risk loci with small individual effects, supporting a polygenic architecture and motivating biologically plausible gene–environment (G×E) investigations (2).

Among environmental risks, infections - particularly during prenatal and early developmental windows - have been repeatedly linked to psychosis (3). One pathogen of sustained interest is *Toxoplasma gondii* (TG), an obligate intracellular protozoan with neurotropism and lifelong latency in neural and muscle tissue. Up to one-third of the world’s population is seropositive, with geographic variation by climate, diet, and feline exposure. While acute infection in immunocompetent hosts is often asymptomatic, converging evidence suggests that latent infection may subtly influence neurobiology and behavior.

Animal studies provide experimental plausibility. TG-infected rodents lose innate aversion to feline odors while maintaining normal responses to other predators, increasing predation risk and facilitating parasite transmission to cats (4). Parasite cysts localize to limbic circuits implicated in fear and salience. Biochemically, infected rodents show increased dopamine (5), while antipsychotics and mood stabilizers can inhibit TG replication and partially reverse parasite-induced behaviors (6), highlighting dopaminergic and limbic pathways central to psychosis.

Human data examining the association between Toxoplasma gondii (TG) infection and schizophrenia remain heterogeneous. A meta-analysis of 23 case–control studies reported an odds ratio of approximately 2.7 for TG seropositivity in schizophrenia, yet subsequent cohort and population-based studies have yielded null or attenuated associations across settings (7). More recent systematic reviews emphasize substantial between-study variability related to differences in serological assays and thresholds, background population seroprevalence, diagnostic definitions, and sampling strategies, limiting the interpretability of adult binary serostatus as a stand-alone risk marker (9). Importantly, meta-analytic syntheses across broader psychiatric and neurological disorders suggest that TG seroprevalence is not specific to psychosis and varies considerably across phenotypes and study designs (10). Contemporary immunopsychiatry models therefore increasingly conceptualize infection-related risk as emerging from interactions between immune regulation, genetic susceptibility, and environmental exposures rather than pathogen exposure alone (11, 12).

Within this framework, adult TG IgG seropositivity may represent a distal and biologically coarse proxy of exposure. This limitation motivates mechanistically informed gene–environment approaches incorporating host immune context and developmental timing. Critically, adult serostatus incompletely indexes exposure timing, which may be most consequential during prenatal or early developmental periods (3). Beyond categorical diagnoses, TG has been linked to altered motor performance, accident risk, and personality traits such as heightened suspiciousness in males and reduced novelty-seeking, though findings vary (8).

These limitations have shifted attention toward host immune mechanisms that may moderate infection-related risk rather than pathogen exposure alone. Host immunogenetic variation may condition vulnerability. Interferon-gamma (IFN-γ) is pivotal for intracellular pathogen control, mediating microglial/macrophage activation, antigen presentation, and growth restriction and stage conversion of TG (13). A functional intronic polymorphism, IFN-γ +874T/A, correlates with cytokine production (14). Reduced IFN-γ production has been repeatedly observed in schizophrenia, including at baseline and under antipsychotic treatment, consistent with a Th2 shift and attenuated cellular immunity (15). Decreased IFN-γ secretion among unaffected relatives in multiplex families suggests a heritable immune endophenotype linked to vulnerability (16). The +874 A allele has been associated with lower IFN-γ output, increased susceptibility to infections such as tuberculosis (17), and, in some psychiatric cohorts, with paranoid schizophrenia in males (18).

Together, these observations motivate a G×E model: genetically reduced IFN-γ could permit low-level TG activity in neural tissue, perturb dopaminergic signaling, and increase risk for paranoid schizophrenia, potentially in a sex-specific manner. Behavioral correlates in TG-seropositive males further reinforce this hypothesis (8). Yet few studies explicitly modeled TG serostatus, IFN-γ +874T/A genotype, and their interaction within a diagnostically and demographically homogeneous cohort. We therefore tested whether TG serostatus, IFN-γ +874T/A genotype, and their interaction predict paranoid schizophrenia among adult males. By restricting analyses to a single clinical subtype and sex, we aimed to reduce diagnostic heterogeneity and enhance power to detect biologically specific effects. We pre-specified quality controls (HWE in controls), age adjustment, and sensitivity analyses across genetic encodings and age parameterizations.

## Methods

### Study design, setting, and ethical oversight

We performed a case–control study at the Beer Yaakov and Ness-Ziona Mental Health Centers (Israel). Cases were recruited from inpatient and outpatient services; controls were healthy adult males from the same catchment. All participants provided written informed consent, and all procedures adhered to the Declaration of Helsinki. The study was approved by the Beer-Ya’akov–Ness Ziona Mental Health Center IRB (Genetic experiment 042-2013; institutional Helsinki study no. 292) and by the Department for Clinical Trials at the Israeli Ministry of Health.

### Dates

Samples were collected between July 2014 and January 2015.

### Participants and eligibility

Cases were adult males (≥18 years) meeting DSM-IV criteria for paranoid schizophrenia.

Diagnosis was established and confirmed by a board-certified psychiatrist with clinical responsibility within the department, based on structured clinical assessment and comprehensive review of the full psychiatric medical record.

All consecutive eligible clinical files during the sampling period were screened for inclusion, and only patients with a clearly documented DSM-IV diagnosis of paranoid schizophrenia were included.

Controls were adult males (≥18 years) recruited from the same catchment area. All controls underwent clinical screening and medical record review to exclude any current or lifetime psychiatric disorder, including psychotic, mood, anxiety, neurodevelopmental, and substance use disorders, as well as any documented neurological condition. Controls were not receiving antipsychotic or other psychotropic medication at the time of sampling. Accordingly, the control cohort was free of both psychiatric and neurological diagnoses.

The use of DSM-IV paranoid schizophrenia criteria reflects the diagnostic framework operative during the study period. Under contemporary DSM-5 nosology, this subgroup would largely correspond to schizophrenia characterized by predominance of positive symptoms, particularly persecutory delusions and related thought content.

Age data were available for essentially all participants. Detailed clinical covariates (e.g., illness duration, medication dose, comorbidities) were not uniformly present in the final analytic dataset.

### Laboratory assessment of *Toxoplasma gondii*

Serum anti–Toxoplasma gondii (TG) IgG and IgM antibodies were assayed using automated ELFA/VIDAS platforms (bioMérieux) according to manufacturer instructions. The primary exposure variable was binary TG serostatus (positive vs. negative) based on established laboratory thresholds; equivocal results were coded as negative. In a linked subset with available laboratory exports, quantitative serological indices (e.g., index ratio, signal-to-cutoff [S/CO], cut-off index [COI]) were additionally evaluated in exploratory analyses as continuous (per 1 SD) and categorical (quartiles) variables, without materially altering inference. TG seropositivity reflected IgG positivity consistent with latent infection, and no isolated IgM-positive cases were identified.

### Genotyping of IFN-γ +874T/A

Whole blood was collected in EDTA tubes, stored at −20 °C, and processed at a central psychiatric genetics laboratory (Hadassah–Hebrew University Medical Center). Genomic DNA was extracted with Qiagen kits. IFN-γ +874T/A genotypes (AA/TA/TT) were determined using Applied Biosystems^®^ TaqMan^®^ SNP Genotyping Assays following standard protocols. Quality control included testing genotype frequencies among controls for Hardy–Weinberg equilibrium (HWE) with χ².

### Variables and coding

- Outcome: Case (schizophrenia) vs. control (healthy).
- Exposures: TG serostatus (negative/positive); IFN-γ genotype (AA, TA, TT; AA as reference).
- Interaction: TG × genotype term.
- Covariate: Age (years; continuous; sensitivity analyses with quartiles).
- Alternative encodings: Dominant (TT vs. TA+AA), recessive (AA vs. TA+TT), additive (T-allele count).

### Statistical analysis

Primary analyses employed logistic regression modeling case status as a function of TG serostatus, genotype, TG×genotype interaction, and age. Odds ratios (ORs), 95% confidence intervals (CIs), and two-sided p-values (α = 0.05) are reported. Reference categories were TG-negative and genotype AA. Sensitivity analyses examined dominant, recessive, and additive genetic encodings as well as alternative age parameterizations. Hardy–Weinberg equilibrium among controls was evaluated using χ² tests. All analyses were conducted using R (version 4.3.1), and all statistical tests were two-tailed with α = 0.05.

### Sample size and power

The final analytic dataset comprised N = 205 individuals (103 cases, 102 controls), with minor variation across variables (e.g., TG serology available for 99 controls). Given the near-identical TG+ prevalence in cases and controls, realized power to detect small differences in main effects or interactions was limited. Nevertheless, model-based CIs are informative for excluding large interaction effects under these measurement choices.

## Results

### Participants

The analytic sample comprised 103 cases and 102 controls with available age data.Mean age (± SD) was 37.5 ± 12.8 years in cases and 41.4 ± 11.8 years in controls (**Table 1**). At the time of biological sampling, patients were receiving routine antipsychotic treatment as part of standard clinical care. Detailed medication dosage and duration data were not consistently available for inclusion in statistical analyses. Because antipsychotic exposure at sampling does not necessarily reflect exposure at the time of Toxoplasma gondii acquisition, timing-specific medication effects could not be evaluated.

**Table 1:** Sample characteristics by outcome.

Outcome	N	Age, mean ± SD (y)	TG seropositive (%)
Case	103	37.5 ± 12.8	17.5 (18/103)
Control	102	41.4 ± 11.8	18.2 (18/99)

TG% is among those assayed for TG.

### Toxoplasma gondii serostatus

TG serology was available for 103/103 cases and 99/102 controls. TG seropositivity was 17.5% (18/103) in cases vs. 18.2% (18/99) in controls (**Table 1**). Within genotype strata, TG+ proportions were similar between cases and controls (**Table 2**).

**Table 2:** TG seropositivity by outcome and genotype.

Outcome	Genotype	TG+ (n)/N	TG+ (%)
Case	AA	5/17	29.4
Case	TA	8/54	14.8
Case	TT	5/32	15.6
Control	AA	5/23	21.7
Control	TA	8/51	15.7
Control	TT	5/25	20.0

### IFN-γ +874T/A genotype distributions and HWE

Genotype data were available for 103 cases and 99 controls. Among controls, counts were AA = 23, TA = 51, TT = 25 (**Table 3**). Control genotypes conformed to HWE (χ² = 0.093, p = 0.760). Among cases, genotype counts were AA = 17, TA = 54, TT = 32 (**Table 3**).

**Table 3:** IFN-γ +874T/A genotype distribution by outcome (counts).

Outcome	AA	TA	TT	Total (genotyped)
Case	17	54	32	103
Control	23	51	25	99

Hardy–Weinberg equilibrium among controls: χ² = 0.093, p = 0.760 (n = 99).

### Association of TG, genotype, and TG×genotype with case status

In the primary logistic model (TG, genotype, TG×genotype, age), TG seropositivity was not significantly associated with case status (**Table 4**), and no TG×genotype interaction was detected. Although the adjusted odds ratio for TG seropositivity was numerically elevated (OR = 1.96), the estimate was highly imprecise (95% CI: 0.45–8.55). Given the virtually identical crude TG seroprevalence between cases and controls (17.5% vs. 18.2%), this elevation likely reflects model instability due to sparse cell counts rather than a meaningful association. Adjusting for age did not materially alter conclusions.

**Table 4:** Logistic regression (outcome ~ TG + genotype + TG×genotype + age).

Term	OR	95% CI	P
TG positive (vs negative)	1.96	0.45–8.55	0.368
TA (vs AA)	1.66	0.70–3.95	0.252
TT (vs AA)	2.09	0.82–5.35	0.122
TG×TA	0.62	0.10–3.74	0.598
TG×TT	0.58	0.11–2.91	0.506
Age (per year)	0.98	0.95–1.00	0.057

Reference categories: TG-negative; genotype AA.

### Sensitivity analyses

**Figure 1:**
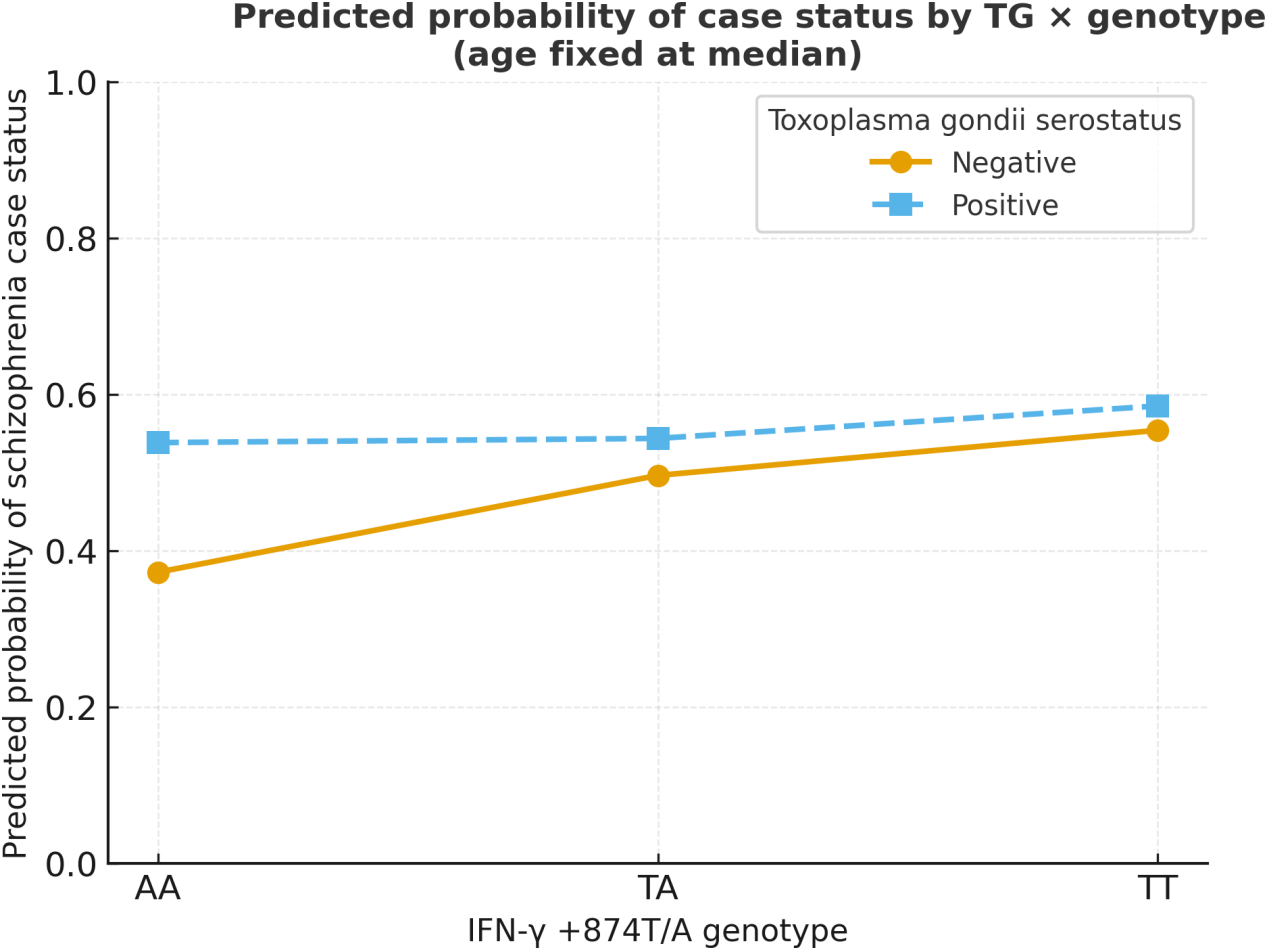
Predicted probability of schizophrenia case status by TG serostatus and IFN-γ +874T/A genotype (age fixed at median). Logistic regression–based predicted probabilities are plotted across genotypes (AA, TA, TT) with separate lines for TG− and TG +. Lines overlap closely across genotypes, visually reinforcing the null interaction. Confidence intervals for predicted probabilities were wide and overlapped substantially. For clarity of visualization, only point estimates are shown.

Results were consistent across dominant, recessive, and additive genetic encodings and when age was modeled by quartiles. In the lab-linked subset, continuous serology (per 1 SD and by quartiles; threshold at index = 1.0 where applicable) did not materially change inference. Collectively, these findings argue against large G×E effects in this cohort under the exposure and genotype operationalizations used in the present study. **Figure 1** presents the predicted probability of case status by TG serostatus and genotype, illustrating the absence of a meaningful interaction effect.

## Discussion

This study tested a mechanistically grounded gene–environment (G×E) hypothesis at the interface of infection biology and psychiatric genetics. In a clinically homogeneous cohort of adult males with paranoid schizophrenia, we found no evidence for a main effect of Toxoplasma gondii serostatus, no independent effect of IFN-γ +874T/A genotype, and no TG×genotype interaction. Genotype distributions among controls conformed to Hardy–Weinberg equilibrium, supporting genotyping quality. Sensitivity analyses using multiple genetic encodings (dominant, recessive, additive) and alternative age parameterizations (continuous, quartiles) produced convergent null findings.

### Relation to prior evidence

Our results differ from meta-analytic estimates suggesting elevated TG antibody prevalence in schizophrenia (7), and from reports of male-specific associations between IFN-γ +874T/A and paranoid schizophrenia (14). At the same time, they align with other studies failing to detect robust TG–schizophrenia associations once methodological variability is accounted for. It is plausible that previously observed effects reflected assay heterogeneity, population structure, unmeasured confounding, or publication bias favoring positive findings.

The animal literature offers strong biological plausibility—TG-induced behavioral specificity (4), dopaminergic alterations (5), and pharmacologic sensitivity to antipsychotics (6) - yet translation to human psychosis may depend on timing, infection burden, parasite strain, and host immune context. These factors are not well captured by adult binary serostatus or a single immune polymorphism.

It should also be noted that rs2430561 represents an indirect proxy for IFN-γ–related immune activity rather than a confirmed causal regulatory variant (14). The functional architecture of the IFNG locus remains incompletely characterized, and population-specific linkage disequilibrium patterns around IFNG may influence how well rs2430561 captures functional variation across different ancestral groups. Differences in haplotype structures between populations may therefore contribute to inconsistencies in reported associations. In addition, large-scale schizophrenia GWAS conducted by the Psychiatric Genomics Consortium (2) have not identified strong genome-wide significant signals at the IFNG locus, suggesting that any immune-related genetic effects involving IFNG are likely modest or context-dependent.

In this context, it is important to emphasize that IFN-γ +874T/A represents an indirect genetic proxy of immune function rather than a direct measure of cytokine activity or cellular immune responsiveness. While the polymorphism has been associated with variability in IFN-γ production, immune signaling is dynamic and influenced by multiple genetic, developmental, and environmental factors. Accordingly, conclusions regarding impaired IFN-γ–mediated immunity in schizophrenia should be interpreted cautiously, particularly in adult, clinically treated populations.

### Potential explanations for null findings

Several non-mutually exclusive explanations may account for the null findings observed in this study. First, adult TG serostatus reflects cumulative lifetime exposure but does not capture the developmental timing of infection, which may be most etiologically relevant during prenatal or early-life periods. Second, binary serostatus is a biologically coarse proxy that does not incorporate antibody titers, avidity, parasite burden, or strain variation, potentially obscuring more subtle host–pathogen effects. Third, schizophrenia is highly polygenic, and single functional immune polymorphisms are likely to exert modest and context-dependent influences within broader immune-genetic architectures.

Importantly, given the relatively low prevalence of TG seropositivity (~18%) and stratification across three genotype categories, several TG–genotype cells were necessarily small. While the present data allow exclusion of large interaction effects under the exposure and genotype definitions used here, smaller to moderate effects cannot be ruled out. The wide confidence intervals surrounding interaction estimates therefore likely reflect statistical imprecision, a common limitation in candidate gene–environment studies in psychiatry.

### Strengths and limitations

This study has several strengths, including a pre-specified and biologically grounded gene–environment hypothesis, restriction to a clinically homogeneous subgroup (male patients with paranoid schizophrenia), use of validated serological and genotyping assays with confirmation of Hardy–Weinberg equilibrium, and consistent findings across multiple sensitivity analyses. These design features enhance internal validity and reduce diagnostic heterogeneity commonly affecting infection-related psychiatric research.

Several limitations should also be considered. The case–control design precludes causal inference, and reliance on binary TG serostatus rather than quantitative antibody measures or avidity indices may have limited biological resolution. Environmental and clinical covariates, including detailed exposure history, illness duration, and medication dosage, were not uniformly available for inclusion in analytic models.

Most patients were receiving routine antipsychotic treatment at the time of biological sampling. Antipsychotic medications have documented immunomodulatory properties and may influence host immune signaling or parasite–host interactions, potentially attenuating observable associations, although established IgG serostatus itself is unlikely to be directly altered. The absence of detailed medication variables in the analytic models therefore represents an additional limitation and may have attenuated immune-related associations.

Finally, given the sample size and TG seropositivity prevalence, statistical power was sufficient to exclude large interaction effects under the exposure and genotype definitions used here but remained limited for detecting small to moderate effects.

### Implications and future directions

Our findings constrain the plausible magnitude of TG–IFN-γ (+874T/A) effects in male paranoid schizophrenia under common operationalizations, and discourage overreliance on single-SNP × single-exposure models. Publishing rigorous null results refines effect-size expectations, reduces research waste, and aids meta-analytic synthesis. Future research should:

- Emphasize developmental timing: Prospective birth-cohort studies integrating maternal serology, immune markers, and placental pathology.
- Incorporate quantitative immunology: Standardized antibody titers, avidity, subclasses, antigenemia/PCR, and TG strain typing.
- Broaden genetics: Immune-gene panels, polygenic risk scores, HLA/MHC and complement, microglial activation pathways; G×E×D (Gene × Environment × Development) frameworks.
- Harmonize across cohorts: Standardized assays, thresholds, and pre-registered protocols to enable individual-participant-data meta-analyses.
- Leverage endophenotypes: Motor function, cognition, and sensory processing may be more sensitive to TG/immune variation than categorical diagnosis.
- Explore neuroinflammatory pathways: Integrating infection hypotheses with broader models of microglial and neuroimmune dysfunction.

## Conclusion

Despite strong biological plausibility, our findings do not support the presence of a large TG–IFN-γ (+874T/A) interaction in adult male paranoid schizophrenia under the exposure and genotype definitions used in this study. Smaller interaction effects, however, cannot be excluded given the available sample size and exposure prevalence. Transparent reporting of null findings contributes to refining effect-size expectations in infection-related models of psychosis and may help reduce publication bias in candidate gene–environment research.

From a clinical perspective, these results caution against overinterpreting adult TG seropositivity as a biomarker of psychosis risk. At the public-health level, the findings highlight the need for more precise developmental and immunological markers before infection-related prevention or screening strategies can be justified.

## Data Availability

The datasets presented in this article are not publicly deposited due to ethical and institutional constraints. Data are available from the corresponding author upon reasonable request.
