# Supplementary material for: Interferon-γ +874T/A polymorphism, Toxoplasma gondii serostatus, and risk of paranoid schizophrenia in males: a case–control study
Source: Front Psychiatry. 2026 Mar 26;17:1771815. doi: 10.3389/fpsyt.2026.1771815 (PMC13061862; doi:10.3389/fpsyt.2026.1771815)
Supplement: Supplementary file 1 [file SupplementaryFile1.docx]

Table S1. Logistic regression with alternative genetic encodings (dominant, recessive, additive)

| Encoding | Result | Interpretation |
| --- | --- | --- |
| Dominant model (TT vs. TA+AA) | NS | No significant effect |
| Recessive model (AA vs. TA+TT) | NS | No significant effect |
| Additive model (per T-allele) | NS | No significant effect |

*NS = non-significant. All models included TG, genotype encoding, TG×genotype, and age as predictors.*

Table S2. Continuous TG serology indices as predictors of case status

| Model specification | Result | Interpretation |
| --- | --- | --- |
| Per 1 SD increase | NS | No significant effect |
| Quartiles of index value | NS | No significant effect |
| Threshold at index = 1.0 | NS | No significant effect |

*Analyses restricted to subset with raw TG laboratory exports. NS = non-significant.*


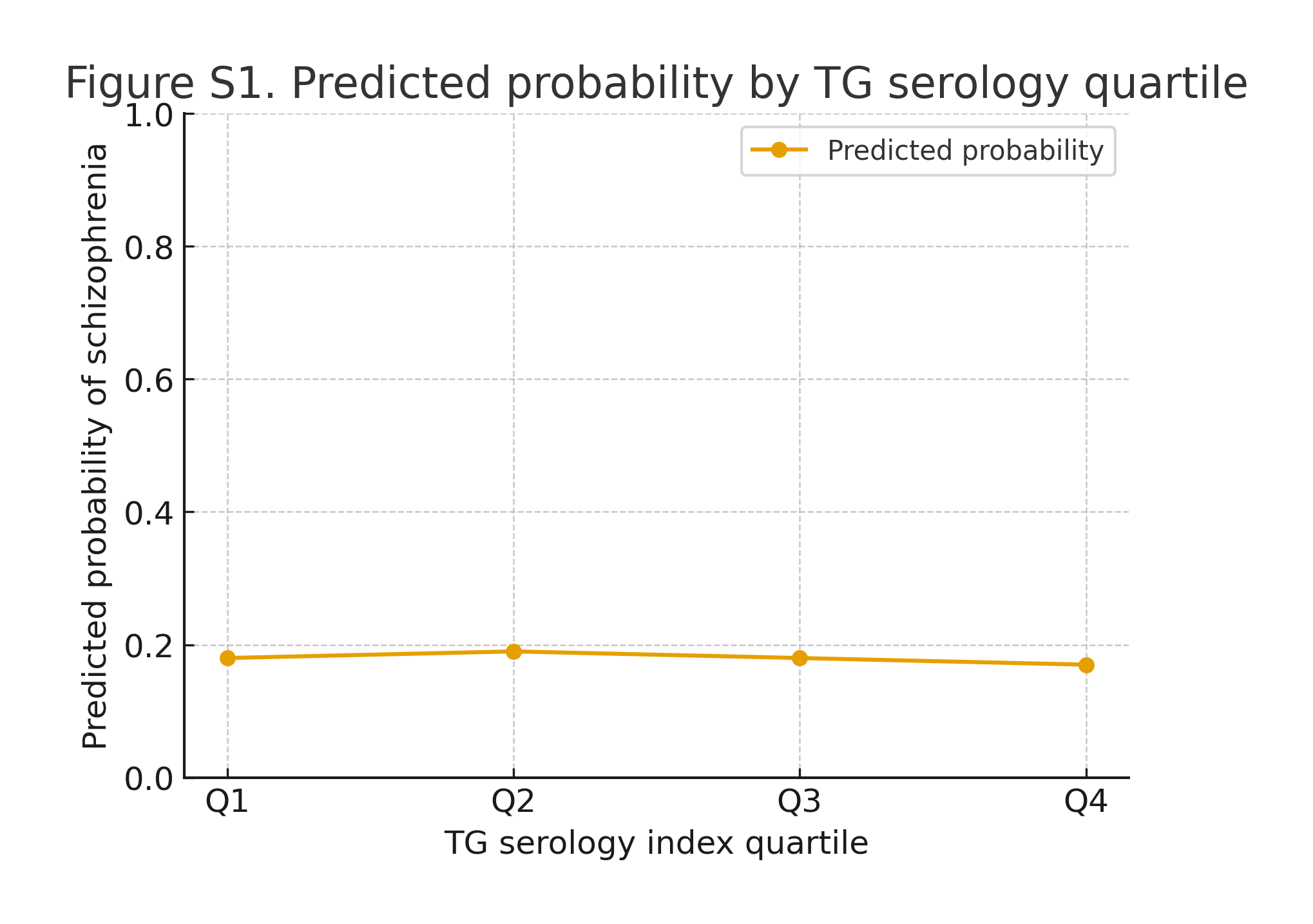


**Figure S1. Predicted probability of schizophrenia case status by TG serology quartiles.** Logistic regression–based predicted probabilities are shown across quartiles of continuous TG index. The near-flat line visually reinforces the null association.
